# CDO, an Hh-Coreceptor, Mediates Lung Cancer Cell Proliferation and Tumorigenicity through Hedgehog Signaling

**DOI:** 10.1371/journal.pone.0111701

**Published:** 2014-11-04

**Authors:** Young-Eun Leem, Hye-Lim Ha, Ju-Hyeon Bae, Kwan-Hyuck Baek, Jong-Sun Kang

**Affiliations:** Department of Molecular Cell Biology, Sungkyunkwan University School of Medicine, Samsung Biomedical Research Institute, Suwon, 440–746, Republic of Korea; Cincinnati Children's Hospital Medical Center, United States of America

## Abstract

Hedgehog (Hh) signaling plays essential roles in various developmental processes, and its aberrant regulation results in genetic disorders or malignancies in various tissues. Hyperactivation of Hh signaling is associated with lung cancer development, and there have been extensive efforts to investigate how to control Hh signaling pathway and regulate cancer cell proliferation. In this study we investigated a role of CDO, an Hh co-receptor, in non-small cell lung cancer (NSCLC). Inhibition of Hh signaling by SANT-1 or siCDO in lung cancer cells reduced proliferation and tumorigenicity, along with the decrease in the expression of the Hh components. Histological analysis with NSCLC mouse tissue demonstrated that CDO was expressed in advanced grade of the cancer, and precisely co-localized with GLI1. These data suggest that CDO is required for proliferation and survival of lung cancer cells via Hh signaling.

## Introduction

Hh signaling pathway is one of essential signaling pathways, which is implicated in embryonic development, morphogenesis and proliferation [1], [2], [3], [4], [5], [6]. The molecular mechanism how to regulate Hh signaling is still under investigation. Generally, once Hh ligand binds to its primary receptor, Patched 1 (PTCH1), Smoothened (SMO) is released from PTCH1-mediated inhibition and migrates to primary cilium. Stimulation of SMO triggers sequential signal transduction that activates the transcription factors of GLI family. The active form of GLI protein is translocated into the nucleus and regulates the expression of downstream target genes, including PTCH1 and GLI1 [1], [7], [8].

The loss of Hh signaling during embryonic development is associated with several genetic disorders including holoprosencephaly, which is the most common malformation of the forebrain [9], [10], [11]. In contrast, constitutive activation of Hh signaling has been known to be involved in initiation and progression of several cancers in skin (sporadic basal cell carcinoma, BCC), brain (medulloblastoma), muscle (rhabdomyosarcoma, RMS), gastrointestinal tract, prostate, pancreas, and lung [12], [13], [14], [15], [16], [17], [18], [19], [20], [21], [22], [23]. The link of Hh pathway to carcinogenesis was initially reported in Gorlin syndrome in which the mutation in the *PTCH1* gene is responsible for the cancer incidence [24]. Moreover, the aberrant upregulation of Hh signaling through the loss of PTCH1 or the gain-of-function mutation in *SMO* was extensively studied in BCC and medulloblastoma [13], [14].

The significance of Hh signaling in carcinogenesis was also explored in the proliferation of small cell lung cancer (SCLC), which is a highly aggressive lung cancer constituting about 20–25% of all lung cancers [21]. Inhibition of the activity of Hh signaling using SMO antagonist, cyclopamine resulted in the serious growth reduction in SCLC cell lines [21], [23], [25], [26]. Whereas, it was initially suggested that Hh signaling is less associated with NSCLC, the most dominant type of lung cancer and the most lethal malignancy. However, several evidences have recently indicated that NSCLC is dependent on Hh signaling activity in proliferation as well [27], [28], [29], [30].

Although the major receptor against Hh is PTCH1, there are additional co-receptors assisting the Hh signaling positively, such as CDO, BOC and GAS1 [31], [32], [33], [34], [35]. Hh signaling is involved in various cellular and developmental processes, and consequently tight regulations are absolutely required for the signaling to recognize and control micro-variation in cellular environment. Meanwhile, many lung cancer cell lines are producing various levels of Hh ligand [25], [30]. Even if a low level of Hh ligand is seen in some lung cancer cells, these cells reveal the increase in Hh target gene expression implying upregulation of Hh signaling. Under these circumstances, the presence of Hh co-receptors may contribute to the amplification of the weak extracellular cue in cancer cells in addition to the fine adjustment of Hh signaling during embryogenesis.

Among those co-receptors, CDO is a transmembrane protein belonging to the immunoglobulin (Ig)/fibronectin type III (FNIII) superfamily and plays an important role in muscle differentiation, embryonic development and neuronal differentiation [36], [37], [38], [39]. Structural analysis demonstrated that the fibronectin repeats in the extracellular domain of CDO is critical for Hh binding [39]. The positive regulation of Hh signaling pathway by CDO was initially identified in *Drosophila*. It has been known that ihog (ortholog of CDO in *Drosophila*) together with boi (ortholog of BOC in *Drosophila*) is important to the activation of Hh signaling in the developing wing imaginal disc [40], [41]. In mice, Cdo deficiency exhibits multiple congenital defects, including holoprosencephaly, which is the defect commonly, associated with mutations in Hh signaling [39], [42]. Furthermore, CDO is involved in Hh-mediated ventral neural patterning of the mammalian neural tube [31] and as well as in Hh-mediated cell proliferation in cerebellar granule neuron progenitors (CGNPs) [43].

In addition to the role of CDO in organ development and cellular differentiation, the association of CDO to Hh signaling, which is implicated in tumorigenesis intrigued us to elucidate a role of CDO in lung cancer cell proliferation. In the present study we sought to uncover the contribution of CDO to Hh signaling in NSCLCs and additionally to proliferation and tumorigenicity in lung tumor cells. We found that the level of CDO was higher in NSCLC cells, and that the inhibition of CDO expression downregulated Hh signaling, and reduced proliferation and tumorigenesis in human lung cancer cells. In addition, CDO expression was observed in advanced stage of NSCLC tissues. Taken together, all data suggest that CDO, as a co-receptor for Hh, is crucial for cancer cell proliferation and tumorigenicity, and therefore it can be considered as a good candidate for anticancer therapeutic applications.

## Materials and Methods

### Cell cultures and reagents

BEAS-2B and NSCLC cell lines, A549, H1299, H460 and H520 were kindly provided by Prof. D. H. Kim (Sungkyunkwan University, Samsung Biomedical Research Institute, Korea) [44]. The culture of BEAS-2B followed ATCC's guidelines. NSCLC cells were cultured in a humidified incubator with 5% CO_2_ in RPMI-1640 medium (Invitrogen) with 10% fetal bovine serum (FBS) supplemented with penicillin and streptomycin. For measuring mRNA/protein expression or cell death in siCDO-transfected cells, the medium was switched to RPMI-1640 medium with 0.5% FBS one day after transfection, and the transfected cells were cultured for 2 days further. 50 µM of SANT1 (S4572, Sigma-Aldrich) dissolved in DMSO or the corresponding volume of the vehicle DMSO was treated to cells grown in RPMI-1640 medium containing 0.5% FBS at indicated time point.

### RNA interference experiments

Each NSCLC cell line was transfected with siCDO using Lipfectamine RNAiMAX reagent (Invitrogen) according to manufacturer's instructions. The following sequences were used as siRNA against CDO. siCDO #1, Sense, 5′-GGAUCUUGGACCCUUAUGUUU-3′, Antisense, 5′- ACAUAAGGGUCCAAGAUCCUU-3′. siCDO #2, Sense, 5′-CUUCAAAGUCGAAUAUAAAUU-3′, Antisense, 5′- UUUAUAUUCGACUUUGAAGUU′. For *in vivo* tumorigenicity assay, A549 cells that stably express small hairpin RNA (shRNA) against CDO were prepared by transfecting with pSuper-puro-shCDO. pSuper-puro-shCDO vector was reported previously [42].

### Total RNA preparation and real-time qRT-PCR

Total RNA was isolated using easyBLUE total RNA extraction kit (iNtRON Biotechnology Inc., Korea), and cDNA was synthesized using PrimeScript RT reagent kit (TAKARA, Japan) according to manufacturer's instructions. Real-time qRT-PCR was performed using SYBR Premix ExTaq kit (TAKARA, Japan) and Thermal Cycler Dice real time system (TP800, TAKARA, Japan) according to manufacturer's instructions. The forward (F) and reverse (R) primers used in this study are listed below. *SHH* F, 5′-CCAAAAAGCTGACCCCTTTA-3′, R, 5′-GCTCCGGTGTTTTCTTCATC-3′; *PTCH1* F, 5′-CAGCACTGGAAAACTCGTCA-3′, R, 5′-TCTGATGAACCACCTCCACA-3′; *GLI1* F, 5′-AAGGGGTTTCTATCCTTCCAGA-3′, R, 5′-TCCTTTATTATCAGGAAACAGTGTCA-3′; *CDO* F, 5′-GGGAAATACATCTGCGAAGC-3′, R, 5′-CTGAGCAGCATCAGGAAGTG-3′; *18S rRNA* F, 5′-GTAACCCGTTGAACCCCATT-3′, R, 5′-CCATCCAATCGGTAGTAGCG-3′. Relative quantification of a gene expression was described as fold of relative changes in mRNA levels compared to a control, using the 2^−ΔΔCt^ equation, [ΔΔCt = ΔCt (target gene)-ΔCt (reference gene)]. 18S rRNA was used as a reference gene.

### Cell proliferation and viability assay

Cells were seeded at a concentration of 5×10^3^ or 1×10^4^ cells per 96-well and cultured in RPMI-1640 containing 0.5% FBS. Trypsinized cells were stained with trypan blue (TB) solution, and the non-stained viable cells were counted. For MTT assay, 20 µl of 5 mg/ml MTT (3-[4,5-dimethylthiazol-2-yl]2,5-diphenyltetrazolium bromide) was added to 5×10^3^ or 1×10^4^ cells per 96-well. After 4 hour-incubation at 37 °C, the culture soup was switched to 200 µl DMSO. The absorbance was measured at 595 nm. For BrdU assay, cells were grown with 10 µM bromodeoxyuridine (BrdU; Sigma-Aldrich Corp.) for 1 hr. Fixed cells by 4% PFA were probed with anti-BrdU antibody (Chemicon international Inc.). Fluorescence was detected by Alexa Fluor 488 goat anti-mouse antibody (Molecular Probe).

### Flow cytometry assay of apoptosis

Apoptosis was measured by using ApoScan Annexin V FITC apoptosis detection kit (BioBud, Korea) and BD FACS CANTO II flow cytometer (BD Biosciences) according to manufacturer's instructions.

### Western blot

Western blot analyses were performed as described previously [37]. Primary antibodies used in this study are listed as below; Cleaved Caspase3 (#9664, Cell Signaling), Caspase9 (sc-81663, Santa Cruz), Cdk2 (sc-6248, Santa Cruz), Cyclin D1 (sc-246, Santa Cruz), Cyclin E (sc25303, Santa Cruz), GAPDH (AbFRONTIER, Korea), p21 (sc-6246), p27 (ab7961, Abcam).

### Colony forming assay

Five thousand of cells were gently blended with 10% FBS RPMI-1640 medium containing 0.35% agarose. The mixture overlaid on the 0.5% bottom agarose with 10% FBS medium in 6-well dishes. The plated cells were incubated at 37°C in humidified incubator for 3 weeks with feeding of medium every second day. The colony formation was visualized with 0.01% crystal violet and analyzed with a dissecting microscope. The quantification of colony number per field was determined by using ImageJ. Each experiment was performed in triplicate and repeated twice.

### *In viv*o tumorigenicity assay

5×10^6^ of the control (pSuper-puro)- or pSuper-puro-shCDO-transfected A549 cells were subcutaneously injected into the flanks of 6- to 8-week-old female nude mice. Tumor volume was measured every 4 days, and calculated as described [45]. All animal studies were reviewed and approved by the International Animal Care and Use Committee (IACUC) of SKKU School of Medicine (SUSM). SUSM is an Association for Assessment and Accreditation of Laboratory Animal Care (AAALAC) international accredited facility and abides by the Institute for Laboratory Animal Research (ILAR) guide.

### Mice and immunohistochemistry

*LSL-K-ras ^G12D^* mice were provided by Dr. Tyler Jacks (MIT Cancer Center) [46], [47]. The paraffin sections from *LSL-K-ras ^G12D^* mice were immunoreacted with 2B3 (mouse ascites against CDO) and anti-GLI1 antibody (1∶100; ab49314, Abcam), and detected using immunofluorescence. Confocal microscopy was performed at SKKU School of Medicine-Microscopy Shared Resource Facility with Zeiss LSM-510 Meta confocal microscope. All animal studies were reviewed and approved by the International Animal Care and Use Committee (IACUC) of SKKU School of Medicine (SUSM). SUSM is an Association for Assessment and Accreditation of Laboratory Animal Care (AAALAC) international accredited facility and abides by the Institute for Laboratory Animal Research (ILAR) guide.

### Statistical analysis

Statistical analyses were performed using Student's *t* test. Data were presented as means ±SD from at least three independent experiments and considered significant when *p* values were <0.05 (*) or <0.01(**).

## Results

### Hh components, which are involved in lung cancer cell proliferation were inhibited by CDO depletion in NSCLCs

**Figure 1:**
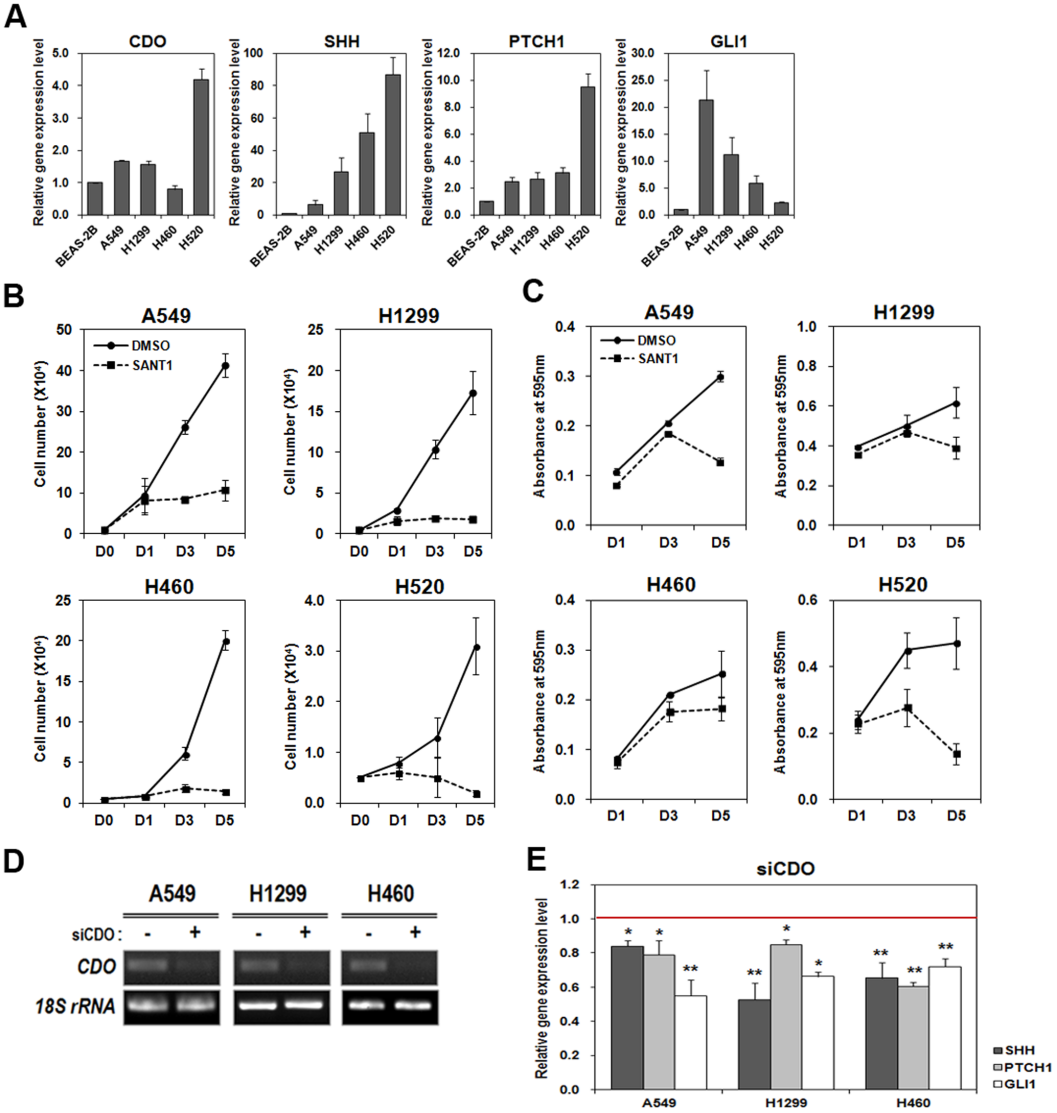
Hh signaling, which is associated with lung cancer cell proliferation was inhibited by CDO depletion. A. Real-time qRT-PCR for the expression levels of CDO and Hh signaling components (SHH, PTCH1 and GLI1) in BEAS-2B and NSCLC cell lines (A549, H1299, H460 and H520). Each expression level was normalized to the level of 18S rRNA. The relative amount of each component in NSCLCs was determined as the amount of each in BEAS-2B was set to 1.0. B. The number of viable cells was determined by cell counting at indicated time in A549, H1299, H460 and H520 treated with or without 50 µM SANT1. C. Cell viability was assessed by MTT assay at indicated time in A549, H1299, H460 and H520 treated with or without 50 µM SANT1. The absorbance was measured at 595 nm. D. RT-PCR for CDO in A549, H1299 and H460 transfected with the scrambled siRNA or siCDO #1. E. Real-time qRT-PCR for Hh signaling components in A549, H1299 and H460 transfected with the scrambled siRNA or siCDO. The expression level of each component was normalized to the level of 18S rRNA. The relative amount of each component in CDO-depleted NSCLCs was determined as the amount of each in the control cells was set to 1.0 (red line). All the values represent means of at least triplicate determinations ±1 SD. *p<0.05 and **p<0.01.

Previous literatures have shown that CDO acts as an accessory receptor for Hh ligand and positively regulates Hh signaling [39], [48]. The close relevance of the activation of Hh signaling to cancer cell proliferation prompted us first to test CDO expression in NSCLC cells. To do so, total RNAs were isolated from A549, H1299, H460 and H520 NSCLC cell lines as well as from BEAS-2B human non-cancerous lung cell, and used for analyzing CDO expression by doing real-time qRT-PCR. The result showed that CDO was expressed higher in A549, H1299 and H520 cells than BEAS-2B however H460 revealed relatively lower CDO level (Figure 1A). In addition, we investigated the expression of Hh signaling components in NSCLC cells by performing real-time qRT-PCR. The higher expression of SHH was seen in all four NSCLC cell lines than in the non-cancerous lung cell. Likewise, the downstream target genes of Hh signaling, PTCH1 and GLI1 were expressed higher in those NSCLC cells (Figure 1A). A549 showed relatively lower level of Hh ligand, compared with other cell lines, however revealed the increase in Hh target gene expression (PTCH1 and GLI1). In H520, CDO, SHH and PTCH1 were expressed comparatively higher than any other cell lines but GLI1 expression was fairly low.

We first determined the inhibitory effect of SANT1 on Hh signaling by examining the expression of Hh signaling target genes after SANT1 treatment. SANT1 binds directly to SMO, impeding the signaling. Total RNAs isolated from DMSO- or SANT1-treated A549, H1299 and H460 were used for analyzing PTCH1 and GLI1 expressions by real-time qRT-PCR. As predicted, the addition of SANT1 resulted in reduction of PTCH1 and GLI1 expressions (Figure S1 in File S1). In order to determine the effect of Hh signaling on the proliferation of NSCLCs, we inhibited Hh signaling in A549, H1299, H460 and H520 by using SANT-1. Then, TB cell counting and MTT assay were performed to analyze cell proliferation and viability. The data showed that the addition of 50 µM SANT1 resulted in significant reduction in cell growth and viability of four individual NSCLC cells (Figure 1B and C). The effect of SANT1-mediated inhibition was stronger at late time points. These data indicate that the tested NSCLC cells require Hh signaling activity for retaining their proliferation.

Next, we asked whether CDO contributed to the activity of Hh signaling in NSCLCs. To determine that, we transfected three different NSCLC cell lines, A549, H1299 and H460 with siCDO or the scrambled siRNA and evaluated the effect of CDO deficiency on the expressions of Hh signaling components by real-time qRT-PCR. The expression of CDO was substantially reduced with siCDO in all cell lines (Figure 1D). To deplete CDO expression, we utilized two different sequences of siRNA, siCDO #1 and #2. Both siCDOs were showing the similar CDO depletion and the results (Data not shown), and we are presenting the data of siCDO #1. The levels of the Hh signaling components, SHH, PTCH1, and GLI1 were significantly reduced in CDO-depleted lung cancer cells compared with the scrambled control (Figure 1E). These data denote that CDO acts as a positive regulator for Hh signaling in NSCLC cells, irrespective of its expression level.

### CDO depletion led to an inhibition of proliferation and an induction of apoptosis in NSCLCs

**Figure 2:**
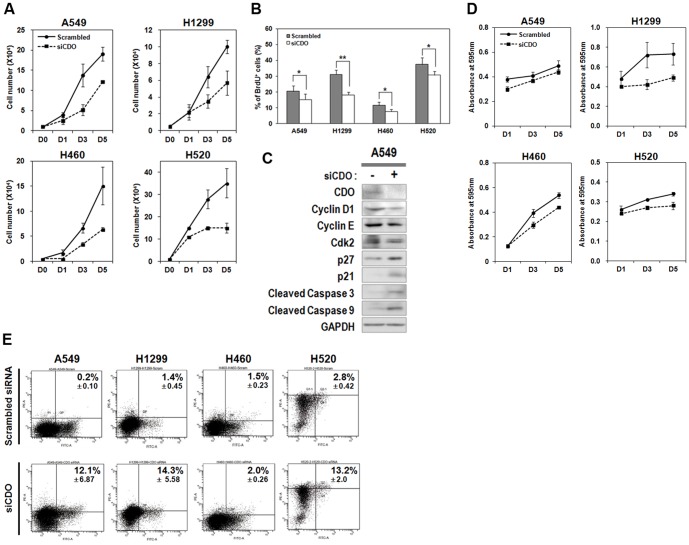
CDO depletion inhibited and promoted cell proliferation and apoptosis in NSCLC cells, respectively. A. The number of viable cells was determined by cell counting at indicated time in the control- or CDO-depleted A549, H1299, H460 and H520. B. Cell proliferation of CDO knockdown A549, H1299, H460 and H520 was determined by BrdU incorporation. C. Western blot analysis for the expression of cell cycle regulators (Cyclin D1, Cyclin E and Cdk2), repressors (p27 and p21) and apoptotic markers (cleaved Caspase 3 and cleaved Caspase 9) in the control or CDO knockdown A549 cells. GAPDH was used as a loading control. D. MTT assay for the cell viability of the control- or siCDO-transfected A549, H1299, H460 and H520 at indicated time. The absorbance was measured at 595 nm. E. Annexin V-FITC apoptosis and flow cytometry analysis with A549, H1299, H460 and H520 transfected with the scrambled siRNA or siCDO. All the values represent means of at least triplicate determinations ±1 SD. *p<0.05 and **p<0.01.

As shown in Figure 1B and C, we observed that proliferation of NSCLC cells was quite sensitive to SANT1. Thus, we decided to determine the effect of the down-regulation of Hh signaling by CDO knockdown on cancer cell proliferation. To do so, we evaluated the rate of cellular proliferation of A549, H1299, H460 and H520 under CDO-depleted condition by performing TB cell counting and MTT assay (Figure 2A and D). The result showed that CDO knockdown in all tested NSCLC cells considerably diminished cell number and viability, compared with the scrambled control. However the extent of the proliferation reduction was not stronger than that in SANT1 treatment (compare Figure 2A and D to Figure 1B and C, respectively). The slope of the graphs for cell number and viability in CDO-depleted cancer cells still kept rising at longer time point day 5, compared with that in SANT1 treatment even though the levels were absolutely much lower than those in the scrambled control cells. To assess the effect of CDO depletion on proliferation further, we performed BrdU incorporation assay. The result showed that CDO knockdown in NSCLC cells led to 20–40% of decrease in cell proliferation (Figure 2B).

The decrease in cell proliferation and viability in the CDO-depleted NSCLC cells could be due to reduced cell cycle progression and/or enhanced cell death. To verify the expression levels of cell cycle regulators/repressors, A549 cells transfected with the scrambled or siCDO were examined for immunoblotting. As a result, the protein levels of cell cycle regulators (Cyclin D1, Cyclin E, and Cdk2) were declined, while the expressions of Cdk inhibitors (p27 and p21) were elevated in siCDO-treated A549 cells compared with the scrambled-treated cells (Figure 2C). These data indicate that the inhibition of CDO expression seems to cause cell cycle arrest in NSCLC cells.

Then, we asked whether CDO depletion was also associated with cell death. Immunoblotting against cleaved forms of caspase-9 and -3 showed that these apoptotic factors were detected stronger in siCDO-transfected A549 than in the control (Figure 2C). Consistently, flow cytometry profile for apoptosis with A549, H1299, H460 and H520 exhibited that higher percentage of annexin-V and PI double positive cells was found in four individual CDO-depleted NSCLCs (Figure 2E). Therefore, these data suggest that the decreased proliferation of NSCLCs upon CDO depletion is due to increased cell death as well as attenuated cell cycle progression.

### Inhibition of CDO expression in NSCLCs showed the reduction of *in vitro* and *in vivo* tumorigenicity

**Figure 3:**
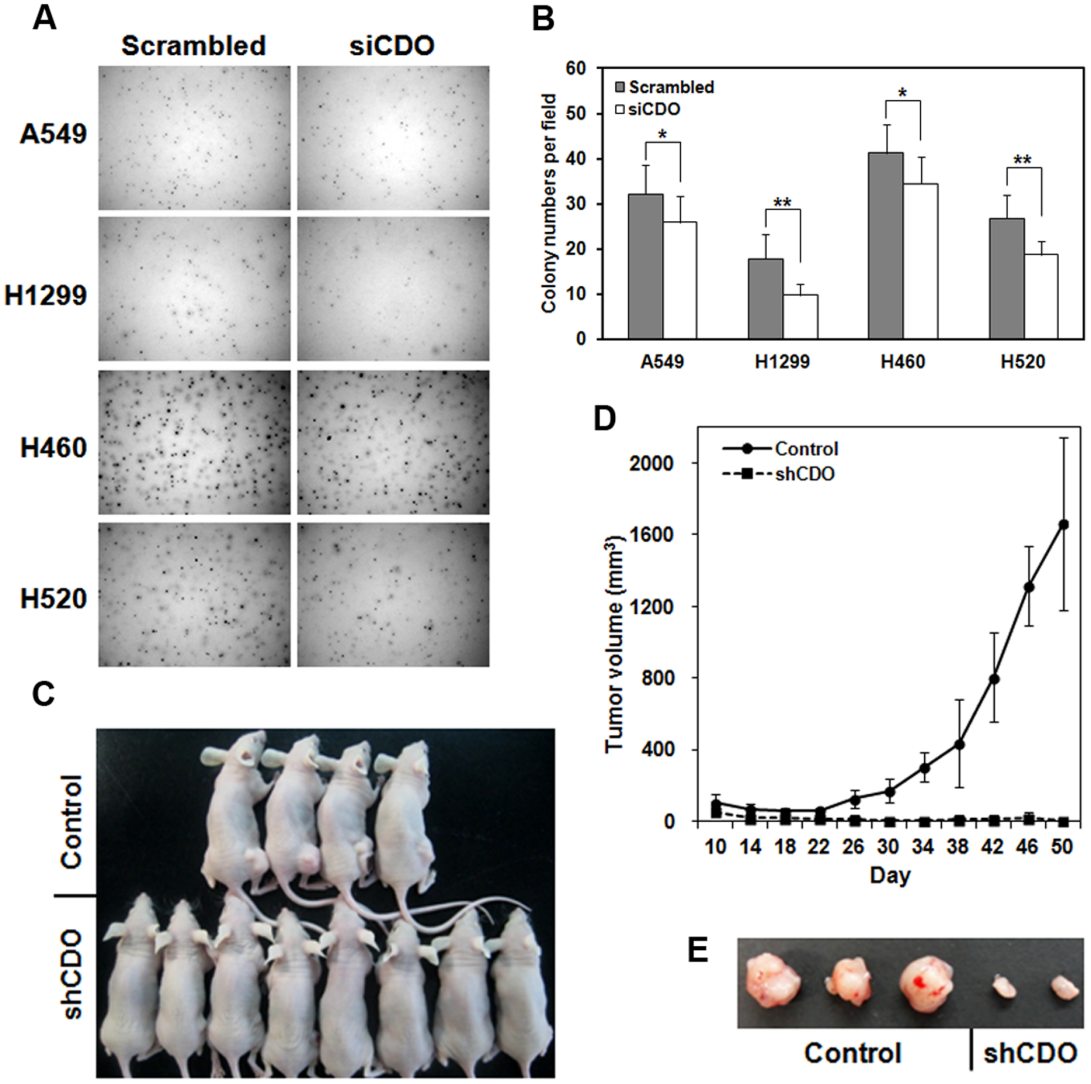
Knockdown of CDO in NSCLCs showed the reduction of *in vitro* and *in vivo* tumorigenesis. A. Colony formation of the control or CDO knockdown A549, H1299, H460 and H520 in soft agar. B. Quantitative analysis of the experiment described in A. Colony numbers per field were determined by ImageJ. The values represent means of triplicate determinations ±1 SD. *p<0.05 and ** *p* <0.01. C. Representative tumor growth 50 days after subcutaneous injection of nude mice with the control or shCDO-treated A549 cells. D. The tumor volume of the nude mice with the control A549 (n = 8) or CDO-depleted A549 (n = 10) described in C. Means are shown as error bars. E. Tumors from the nude mice injected with the control A549 or CDO-depleted A549 cells shown in C.

The diminished cell proliferation in CDO knockdown led us to investigate the influence of CDO depletion on malignant features of NSCLC cells. For that, we assessed the extent of colony formation of siCDO-transfected A549, H1299, H460 and H520 cells in soft agar (Figure 3A and B). The result displayed a marked reduction in clonogenicity *in vitro* when CDO level was decreased.

In order to support the data of *in vitro* tumorigenicity, we prepared stable cell line of A549 expressing shRNA against CDO, and subcutaneously injected the control or CDO-depleted cells to the flanks of nude mice. The tumor size was periodically measured. As shown Figure 3C, D, and E, a significant decrease in tumor growth was observed in nude mice injected with CDO-knockdown A549. Together, these data indicate that CDO is required for the *in vivo* growth of NSCLC cells.

### CDO expression was detected with GLI1 together in advanced stages of NSCLCs

**Figure 4:**
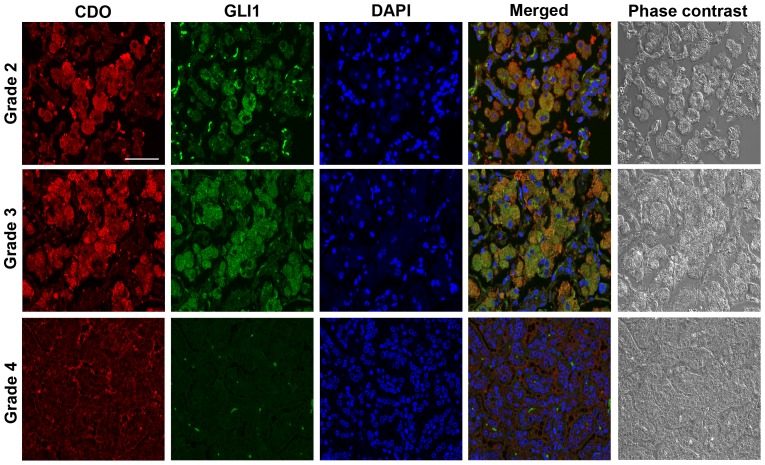
CDO expression was colocalized with GLI1 in high-grade of NSCLCs. Confocal immunofluorescence detection of CDO (red) and GLI1 (green) in grade-2, -3, and -4 lung tumor tissues from *LSL-K-ras ^G12D^* model. Cell nuclei were visualized by DAPI (blue) and the phase contrast images are shown. Scale bar indicates 50 µm.

To determine CDO expression during lung cancer progression, we took advantage of a NSCLC mouse model, *LSL-K-ras^G12D^* mice [46], [47]. After intranasal inhalation of Ad-Cre to induce spontaneous lung tumorigenesis in *LSL-K-ras^G12D^* mice, we assessed CDO expression in the lung tumor tissue showing four individual grades of tumor progression by fluorescent immunohistochemistry. CDO expression was nearly undetectable in grade-1, which is an early stage of tumor progression featuring atypical adenomatous hyperplasia (AAH) or small adenoma (Figure S2 in File S1). However, its expression became enriched as tumor progression has been advanced. In grade-2, in which larger adenomas with a little enlarged nuclei are detected, CDO expression was observed in the cytoplasm of cells in the tumor lesions, and this high expression was retained until grade-3 (Figure 4). As tumors progressed to be invasive adenocarcinomas (grade-4), CDO expression was somewhat decreased and detected in cell membrane. The expression of CDO in the tumor lesions led us to investigate the expression of Hh pathway components in the tumor tissue. Confocal immunofluorescence images showed that GLI1, one of Hh signaling factors was colocalized with CDO, and additionally its expression was also highly observed in grade-2 and -3 tumor lesions, not in grade-4 (Figure 4). The deficiency of GLI1 expression in grade-4 is corresponding to the results suggested by several previous studies [23], [29]. The results of these experiments indicate that CDO expression may be correlated to the up-regulation of Hh signaling in tumor lesions and CDO is also likely related to the progression of lung tumor, not in tumor initiation.

## Discussion

In fact, the significance of CDO in cancer cell proliferation was somewhat uncovered and referred earlier. Hayashi *et al.* [49] made an effort to screen out genes encoding transmembrane proteins, which are highly expressed in prostate cancer cell lines. Among candidates, they focused on 83% of CDO-overexpressed prostate cancer, and demonstrated that reducing CDO expression induced apoptosis and inhibited cell invasion in prostate cancer. However, the molecular mechanism how CDO is involved in proliferation of prostate cancer cells was not explained. In this study, we revealed that the fine-tuning and up-regulation of Hh signaling by CDO is quite required for cell proliferation in lung cancer. We additionally observed that the mRNA level of CDO in the tested lung cancer cell lines except H520 was higher than in BEAS-2B, but it was not significant. Particularly CDO mRNA level in A549 was about 1.7-fold higher than the basal level of it in BEAS-2B. Even though the increase is not dramatically high, it is worthy of notice if we consider that CDO expression is generally found during development and embryogenesis, and hardly detected in adult tissues [36].

It was initially presumed that Hh signaling was rarely linked to NSCLC because Hh and GLI1 were observed at a relatively lower level in NSCLC. Since then, the data, including the present data, of the inhibition of NSCLC proliferation by a SMO antagonist suggested that the activation of Hh signaling via the low level of Hh is certainly associated with tumorigenesis in NSCLC. Under this circumstance CDO may play a critical role as a ligand sensor for a little amount of Hh, supporting the main receptor, PTCH1 and after all induces the activation of Hh signaling.

Meanwhile, the function of CDO in cell proliferation seems to be ambivalent depending on Hh level. Delloye-Bourgeois *et al.* [50] recently suggested that, the Hh level is important for CDO to function as a dependence receptor, which is closely related to the induction of proapoptotic signal. According to them when Hh ligand is fairly limited, caspase 3/9 are recruited to the caspase cleavage site in the intercellular domain of CDO and evoke proteolytic cleavage. This caspase-mediated cleavage eventually triggers apoptosis. The role of CDO as a dependence receptor basically conflicts with the function of CDO in cancer cells with Hh expression, what we uncovered in this study. Therefore, appropriate level of Hh ligand seems to be essential to determine the function of CDO as a positive regulator of Hh signaling or a dependence receptor.

The inhibition of Hh signaling by SANT1 in all NSCLC cells showed more substantial reduction of cell proliferation and viability at longer time point, compared to the inhibition by CDO gene depletion (compare Figure 1B and C to Figure 2A and D, respectively). The limited effect of CDO knockdown on the inhibition of cell proliferation might be due to that other Hh co-receptors, such as BOC and GAS1 are still expressed and active, even though we cannot rule out the possibility of the transient CDO depletion with siRNA. It has been demonstrated that all Hh co-receptors, CDO, BOC, and GAS1 are collectively required for full activation of Hh signaling. CDO, BOC and GAS1 individually make a complex with PTCH1 and the formation of the complexes is critical to Hh-mediated CGNP proliferation [43]. Based on this, understanding the roles of BOC and GAS1 during Hh-mediated tumorigenesis would be needed later.

Detection of CDO expression in high-grade tumor lesion of NSCLC mouse model, not in the lesion of grade-1, excited our curiosity on the role of CDO-mediated Hh signaling in tumor progression. It has been dominantly suggested that the up-regulation of Hh signaling seems to be correlated with tumor grade although it is controversial in some types of cancer. In prostate cancer, the mRNA levels of SHH, PTCH1 and GLI1 were highly elevated in more advanced stages of the cancer, and all those components were closely linked to Gleason score [51]. On the other hand, Gialmanidis *et al.* [29] have immunohistochemically analyzed paraffin-embedded lung tissue sections of 80 NSCLC patients. They found that activation of Hh signaling was associated with tumor grade, and the high activation of the signaling was mainly detected in well-differentiated and moderately differentiated tumors. The robust expression of CDO in the high-grade of tumor may lead to a possibility that CDO as a positive regulator regulates and results in aberrant activation of Hh signaling in tumor development and progression.

So far, the efforts to find anticancer therapies have been targeting mainly SMO, and indeed several SMO inhibitors have been applying to the clinic [52]. The expression of CDO is generally restricted in development and embryogenesis, not in adult stage. Abnormal expression of CDO in the cancerous condition is possibly associated with aberrant cell proliferation. As a part of clinical trials, CDO could be considered as a potential indicator for tumorigenesis and a therapeutic target.

## Supporting Information

File S1 Figure S1, SANT1 inhibited the expression of PTCH1 and GLI1 in NSCLC cells. qRT-PCR for the expression levels of PTCH1 and GLI1 in A549, H1299 and H460, which were treated with DMSO or 50 µM SANT1 for 2 days. Each expression was normalized to the level of 18S rRNA, and the relative amount of each normalized level in SANT1-treated cells was determined as the amount of each in the DMSO-treated cells was set to 1.0 (red line). All the values represent means of triplicate determinations ±1 SD. Figure S2, CDO expression was not detected in grade-1 of NSCLCs. Confocal immunofluorescence detection of CDO (red) in grade-1 lung tumor tissues from *LSL-K-ras ^G12D^* model. Cell nuclei were visualized by DAPI (blue). Scale bar indicates 50 µm.
(DOCX)

## References

[1] Hooper JE, Scott MP (2005) Communicating with Hedgehogs. Nat Rev Mol Cell Biol 6: 306–317. 15803137 10.1038/nrm1622

[2] Jiang J, Hui CC (2008) Hedgehog signaling in development and cancer. Dev Cell 15: 801–812. 19081070 10.1016/j.devcel.2008.11.010 PMC6443374

[3] Joksimovic M, Anderegg A, Roy A, Campochiaro L, Yun B, et al (2009) Spatiotemporally separable Shh domains in the midbrain define distinct dopaminergic progenitor pools. Proc Natl Acad Sci U S A 106: 19185–19190. 19850875 10.1073/pnas.0904285106 PMC2776440

[4] McMahon AP, Ingham PW, Tabin CJ (2003) Developmental roles and clinical significance of hedgehog signaling. Curr Top Dev Biol 53: 1–114. 12509125 10.1016/s0070-2153(03)53002-2

[5] Ingham PW, Placzek M (2006) Orchestrating ontogenesis: variations on a theme by sonic hedgehog. Nat Rev Genet 7: 841–850. 17047684 10.1038/nrg1969

[6] Lum L, Beachy PA (2004) The Hedgehog response network: sensors, switches, and routers. Science 304: 1755–1759. 15205520 10.1126/science.1098020

[7] Ingham PW, McMahon AP (2001) Hedgehog signaling in animal development: paradigms and principles. Genes Dev 15: 3059–3087. 11731473 10.1101/gad.938601

[8] Ogden SK, Ascano M Jr, Stegman MA, Robbins DJ (2004) Regulation of Hedgehog signaling: a complex story. Biochem Pharmacol 67: 805–814. 15104233 10.1016/j.bcp.2004.01.002 PMC3659400

[9] Nanni L, Ming JE, Bocian M, Steinhaus K, Bianchi DW, et al (1999) The mutational spectrum of the sonic hedgehog gene in holoprosencephaly: SHH mutations cause a significant proportion of autosomal dominant holoprosencephaly. Hum Mol Genet 8: 2479–2488. 10556296 10.1093/hmg/8.13.2479

[10] Ming JE, Kaupas ME, Roessler E, Brunner HG, Golabi M, et al (2002) Mutations in PATCHED-1, the receptor for SONIC HEDGEHOG, are associated with holoprosencephaly. Hum Genet 110: 297–301. 11941477 10.1007/s00439-002-0695-5

[11] Roessler E, Du YZ, Mullor JL, Casas E, Allen WP, et al (2003) Loss-of-function mutations in the human GLI2 gene are associated with pituitary anomalies and holoprosencephaly-like features. Proc Natl Acad Sci U S A 100: 13424–13429. 14581620 10.1073/pnas.2235734100 PMC263830

[12] Dahmane N, Lee J, Robins P, Heller P, Ruiz i Altaba A (1997) Activation of the transcription factor Gli1 and the Sonic hedgehog signalling pathway in skin tumours. Nature 389: 876–881. 9349822 10.1038/39918

[13] Xie J, Murone M, Luoh SM, Ryan A, Gu Q, et al (1998) Activating Smoothened mutations in sporadic basal-cell carcinoma. Nature 391: 90–92. 9422511 10.1038/34201

[14] Raffel C, Jenkins RB, Frederick L, Hebrink D, Alderete B, et al (1997) Sporadic medulloblastomas contain PTCH mutations. Cancer Res 57: 842–845. 9041183

[15] Taylor MD, Liu L, Raffel C, Hui CC, Mainprize TG, et al (2002) Mutations in SUFU predispose to medulloblastoma. Nat Genet 31: 306–310. 12068298 10.1038/ng916

[16] Hahn H, Nitzki F, Schorban T, Hemmerlein B, Threadgill D, et al (2004) Genetic mapping of a Ptch1-associated rhabdomyosarcoma susceptibility locus on mouse chromosome 2. Genomics 84: 853–858. 15475264 10.1016/j.ygeno.2004.07.002

[17] Berman DM, Karhadkar SS, Maitra A, Montes De Oca R, Gerstenblith MR, et al (2003) Widespread requirement for Hedgehog ligand stimulation in growth of digestive tract tumours. Nature 425: 846–851. 14520411 10.1038/nature01972

[18] Sheng T, Li C, Zhang X, Chi S, He N, et al (2004) Activation of the hedgehog pathway in advanced prostate cancer. Mol Cancer 3: 29. 15482598 10.1186/1476-4598-3-29 PMC524523

[19] Sanchez P, Hernandez AM, Stecca B, Kahler AJ, DeGueme AM, et al (2004) Inhibition of prostate cancer proliferation by interference with SONIC HEDGEHOG-GLI1 signaling. Proc Natl Acad Sci U S A 101: 12561–12566. 15314219 10.1073/pnas.0404956101 PMC514658

[20] Thayer SP, di Magliano MP, Heiser PW, Nielsen CM, Roberts DJ, et al (2003) Hedgehog is an early and late mediator of pancreatic cancer tumorigenesis. Nature 425: 851–856. 14520413 10.1038/nature02009 PMC3688051

[21] Watkins DN, Berman DM, Burkholder SG, Wang B, Beachy PA, et al (2003) Hedgehog signalling within airway epithelial progenitors and in small-cell lung cancer. Nature 422: 313–317. 12629553 10.1038/nature01493

[22] Watkins DN, Berman DM, Baylin SB (2003) Hedgehog signaling: progenitor phenotype in small-cell lung cancer. Cell Cycle 2: 196–198. 12734424

[23] Chi S, Huang S, Li C, Zhang X, He N, et al (2006) Activation of the hedgehog pathway in a subset of lung cancers. Cancer Lett 244: 53–60. 16446029 10.1016/j.canlet.2005.11.036

[24] Unden AB, Holmberg E, Lundh-Rozell B, Stahle-Backdahl M, Zaphiropoulos PG, et al (1996) Mutations in the human homologue of Drosophila patched (PTCH) in basal cell carcinomas and the Gorlin syndrome: different in vivo mechanisms of PTCH inactivation. Cancer Res 56: 4562–4565. 8840960

[25] Vestergaard J, Pedersen MW, Pedersen N, Ensinger C, Tumer Z, et al (2006) Hedgehog signaling in small-cell lung cancer: frequent in vivo but a rare event in vitro. Lung Cancer 52: 281–290. 16616798 10.1016/j.lungcan.2005.12.014

[26] Park KS, Martelotto LG, Peifer M, Sos ML, Karnezis AN, et al (2011) A crucial requirement for Hedgehog signaling in small cell lung cancer. Nat Med 17: 1504–1508. 21983857 10.1038/nm.2473 PMC3380617

[27] Shi S, Deng YZ, Zhao JS, Ji XD, Shi J, et al (2012) RACK1 promotes non-small-cell lung cancer tumorigenicity through activating sonic hedgehog signaling pathway. J Biol Chem 287: 7845–7858. 22262830 10.1074/jbc.M111.315416 PMC3318742

[28] Bermudez O, Hennen E, Koch I, Lindner M, Eickelberg O (2013) Gli1 mediates lung cancer cell proliferation and Sonic Hedgehog-dependent mesenchymal cell activation. PLoS One 8: e63226. 23667589 10.1371/journal.pone.0063226 PMC3646741

[29] Gialmanidis IP, Bravou V, Amanetopoulou SG, Varakis J, Kourea H, et al (2009) Overexpression of hedgehog pathway molecules and FOXM1 in non-small cell lung carcinomas. Lung Cancer 66: 64–74. 19200615 10.1016/j.lungcan.2009.01.007

[30] Yuan Z, Goetz JA, Singh S, Ogden SK, Petty WJ, et al (2007) Frequent requirement of hedgehog signaling in non-small cell lung carcinoma. Oncogene 26: 1046–1055. 16909105 10.1038/sj.onc.1209860

[31] Allen BL, Tenzen T, McMahon AP (2007) The Hedgehog-binding proteins Gas1 and Cdo cooperate to positively regulate Shh signaling during mouse development. Genes Dev 21: 1244–1257. 17504941 10.1101/gad.1543607 PMC1865495

[32] Martinelli DC, Fan CM (2007) Gas1 extends the range of Hedgehog action by facilitating its signaling. Genes Dev 21: 1231–1243. 17504940 10.1101/gad.1546307 PMC1865494

[33] Okada A, Charron F, Morin S, Shin DS, Wong K, et al (2006) Boc is a receptor for sonic hedgehog in the guidance of commissural axons. Nature 444: 369–373. 17086203 10.1038/nature05246

[34] Seppala M, Depew MJ, Martinelli DC, Fan CM, Sharpe PT, et al (2007) Gas1 is a modifier for holoprosencephaly and genetically interacts with sonic hedgehog. J Clin Invest 117: 1575–1584. 17525797 10.1172/JCI32032 PMC1868789

[35] Tenzen T, Allen BL, Cole F, Kang JS, Krauss RS, et al (2006) The cell surface membrane proteins Cdo and Boc are components and targets of the Hedgehog signaling pathway and feedback network in mice. Dev Cell 10: 647–656. 16647304 10.1016/j.devcel.2006.04.004

[36] Kang JS, Gao M, Feinleib JL, Cotter PD, Guadagno SN, et al (1997) CDO: an oncogene-, serum-, and anchorage-regulated member of the Ig/fibronectin type III repeat family. J Cell Biol 138: 203–213. 9214393 10.1083/jcb.138.1.203 PMC2139939

[37] Kang JS, Bae GU, Yi MJ, Yang YJ, Oh JE, et al (2008) A Cdo-Bnip-2-Cdc42 signaling pathway regulates p38alpha/beta MAPK activity and myogenic differentiation. J Cell Biol 182: 497–507. 18678706 10.1083/jcb.200801119 PMC2500135

[38] Oh JE, Bae GU, Yang YJ, Yi MJ, Lee HJ, et al (2009) Cdo promotes neuronal differentiation via activation of the p38 mitogen-activated protein kinase pathway. FASEB J 23: 2088–2099. 19244314 10.1096/fj.08-119255 PMC2704588

[39] Bae GU, Domene S, Roessler E, Schachter K, Kang JS, et al (2011) Mutations in CDON, encoding a hedgehog receptor, result in holoprosencephaly and defective interactions with other hedgehog receptors. Am J Hum Genet 89: 231–240. 21802063 10.1016/j.ajhg.2011.07.001 PMC3155179

[40] Lum L, Yao S, Mozer B, Rovescalli A, Von Kessler D, et al (2003) Identification of Hedgehog pathway components by RNAi in Drosophila cultured cells. Science 299: 2039–2045. 12663920 10.1126/science.1081403

[41] Camp D, Currie K, Labbe A, van Meyel DJ, Charron F (2010) Ihog and Boi are essential for Hedgehog signaling in Drosophila. Neural Dev 5: 28. 21044292 10.1186/1749-8104-5-28 PMC2984377

[42] Zhang W, Kang JS, Cole F, Yi MJ, Krauss RS (2006) Cdo functions at multiple points in the Sonic Hedgehog pathway, and Cdo-deficient mice accurately model human holoprosencephaly. Dev Cell 10: 657–665. 16647303 10.1016/j.devcel.2006.04.005

[43] Izzi L, Levesque M, Morin S, Laniel D, Wilkes BC, et al (2011) Boc and Gas1 each form distinct Shh receptor complexes with Ptch1 and are required for Shh-mediated cell proliferation. Dev Cell 20: 788–801. 21664577 10.1016/j.devcel.2011.04.017 PMC3432913

[44] Hwang JA, Kim Y, Hong SH, Lee J, Cho YG, et al (2013) Epigenetic inactivation of heparan sulfate (glucosamine) 3-o-sulfotransferase 2 in lung cancer and its role in tumorigenesis. PLoS One 8: e79634. 24265783 10.1371/journal.pone.0079634 PMC3827134

[45] Park BH, Vogelstein B, Kinzler KW (2001) Genetic disruption of PPARdelta decreases the tumorigenicity of human colon cancer cells. Proc Natl Acad Sci U S A 98: 2598–2603. 11226285 10.1073/pnas.051630998 PMC30184

[46] DuPage M, Dooley AL, Jacks T (2009) Conditional mouse lung cancer models using adenoviral or lentiviral delivery of Cre recombinase. Nat Protoc 4: 1064–1072. 19561589 10.1038/nprot.2009.95 PMC2757265

[47] Shin J, Lee JC, Baek KH (2014) A single extra copy of Dscr1 improves survival of mice developing spontaneous lung tumors through suppression of tumor angiogenesis. Cancer Lett 342: 70–81. 24051307 10.1016/j.canlet.2013.08.047

[48] Allen BL, Song JY, Izzi L, Althaus IW, Kang JS, et al (2011) Overlapping roles and collective requirement for the coreceptors GAS1, CDO, and BOC in SHH pathway function. Dev Cell 20: 775–787. 21664576 10.1016/j.devcel.2011.04.018 PMC3121104

[49] Hayashi T, Oue N, Sakamoto N, Anami K, Oo HZ, et al (2011) Identification of transmembrane protein in prostate cancer by the Escherichia coli ampicillin secretion trap: expression of CDON is involved in tumor cell growth and invasion. Pathobiology 78: 277–284. 21849809 10.1159/000329588

[50] Delloye-Bourgeois C, Gibert B, Rama N, Delcros JG, Gadot N, et al (2013) Sonic Hedgehog promotes tumor cell survival by inhibiting CDON pro-apoptotic activity. PLoS Biol 11: e1001623. 23940460 10.1371/journal.pbio.1001623 PMC3735457

[51] Gonnissen A, Isebaert S, Haustermans K (2013) Hedgehog signaling in prostate cancer and its therapeutic implication. Int J Mol Sci 14: 13979–14007. 23880852 10.3390/ijms140713979 PMC3742228

[52] Scales SJ, de Sauvage FJ (2009) Mechanisms of Hedgehog pathway activation in cancer and implications for therapy. Trends Pharmacol Sci 30: 303–312. 19443052 10.1016/j.tips.2009.03.007

