# Supplementary material for: CDO, an Hh-Coreceptor, Mediates Lung Cancer Cell Proliferation and Tumorigenicity through Hedgehog Signaling
Source: PLoS One. 2014 Nov 4;9(11):e111701. doi: 10.1371/journal.pone.0111701 (PMC4219762; doi:10.1371/journal.pone.0111701)
Supplement: File S1 — Figure S1, SANT1 inhibited the expression of PTCH1 and GLI1 in NSCLC cells. qRT-PCR for the expression levels of PTCH1 and GLI1 in A549, H1299 and H460, which were treated with DMSO or 50 µM SANT1 for 2 days. Each expression was normalized to the level of 18S rRNA, and the relative amount of each normalized level in SANT1-treated cells was determined as the amount of each in the DMSO-treated cells was set to 1.0 (red line). All the values represent means of triplicate determinations ±1 SD. Figure S2, CDO expression was not detected in grade-1 of NSCLCs. Confocal immunofluorescence detection of CDO (red) in grade-1 lung tumor tissues from LSL-K-ras G12D model. Cell nuclei were visualized by DAPI (blue). Scale bar indicates 50 µm. (DOCX) [file pone.0111701.s001.docx]

**
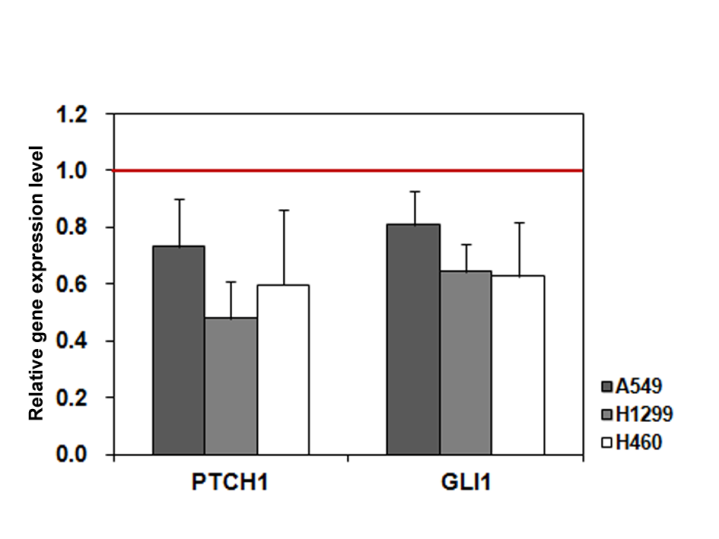
**

**Figure S1. SANT1 inhibited the expression of PTCH1 and GLI1 in NSCLC cells.**

qRT-PCR for the expression levels of PTCH1 and GLI1 in A549, H1299 and H460, which were treated with DMSO or 50µM SANT1 for 2 days. Each expression was normalized to the level of 18S rRNA, and the relative amount of each normalized level in SANT1-treated cells was determined as the amount of each in the DMSO-treated cells was set to 1.0 (red line). All the values represent means of triplicate determinations ±1 SD.


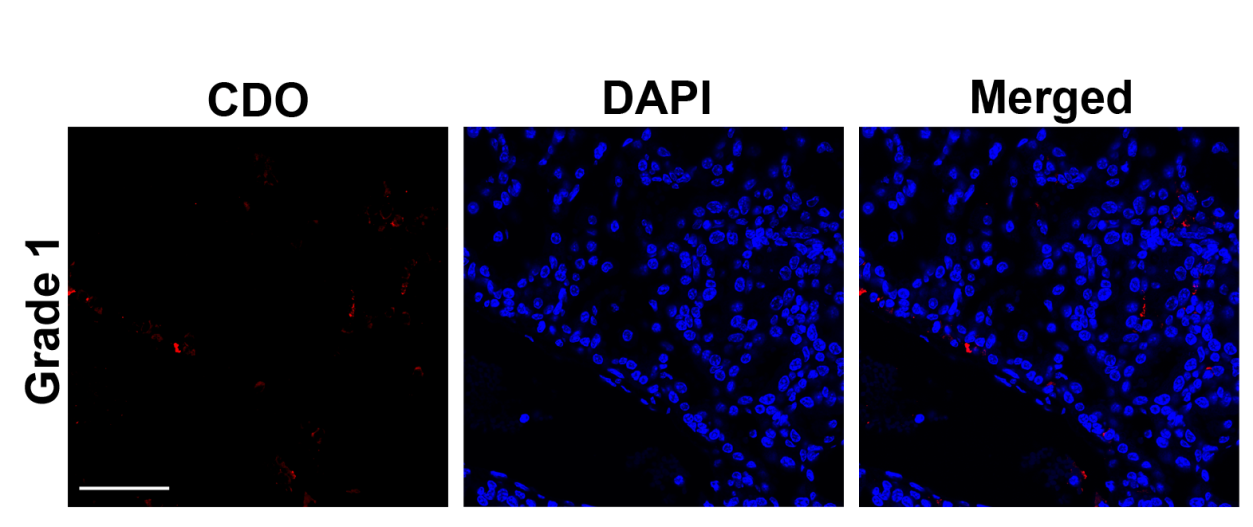


**Figure S2. CDO expression was not detected in grade-1 of NSCLCs.**

Confocal immunofluorescence detection of CDO (red) in grade-1 lung tumor tissues from *LSL-K-ras ^G12D^* model. Cell nuclei were visualized by DAPI (blue). Scale bar indicates 50µm.
